# Feasibility of a multidisciplinary group videoconferencing approach for chronic low back pain: a randomized, open-label, controlled, pilot clinical trial (EN-FORMA)

**DOI:** 10.1186/s12891-023-06763-6

**Published:** 2023-08-09

**Authors:** Iago Garreta-Catala, Rosa Planas-Balagué, Reza Abouzari, Thiago Carnaval, Joan M. Nolla, Sebastián Videla, José-Luis Agulló-Ferré, the EN-FORMA study group, Paula Calvis-Garcia, João Carmezim, Anna Company-Llimona, Esmeralda Fernandez-Mariscal, Coral Fernandez-Solana, Montserrat Figuerola-Batista, Neus Gerique-Fornas, Encarna Grifell-Martín, Nuria Gutierrez-Jimenez, Nuria Mariano-Martin, Xavier Mas-Garriga, Aurema Otero-Gonzalez, Sandra Soler-Soto, Cristian Tebé, Teresa Vázquez-Ventura, Carlota Vázquez-Vera, Ramón Vicent-Porquet

**Affiliations:** 1 Orthopedic Surgery and Traumatology Department, Bellvitge University Hospital, L’Hospitalet de Llobregat, Barcelona, Spain; 2 Rehabilitation and Physical Medicine Department, Bellvitge University Hospital, L’Hospitalet de Llobregat, Barcelona, Spain; 3 Rehabilitation and Physical Medicine Department, Delta del Llobregat Primary Care Center, Barcelona, El Prat de Llobregat Spain; 4 Pharmacology Unit, Department of Pathology and Experimental Therapeutics, School of Medicine and Health Sciences, IDIBELL, Barcelona University, Barcelona, Spain; 5 Rheumatology Department, Bellvitge University Hospital, L’Hospitalet de Llobregat, Barcelona, Spain; 6 Clinical Research Support Unit, Clinical Pharmacology Department, Bellvitge University Hospital, L’Hospitalet de Llobregat, Barcelona, Spain

**Keywords:** Chronic low back pain, Group videoconferencing, Multidisciplinary approach, Lumbar surgery, Psychosocial

## Abstract

**Background:**

Low back pain is a common condition that becomes even more prevalent with aging. A non-pharmacological multidisciplinary approach for chronic non-specific low back pain (CNSLBP) has been recommended, but integrating different healthcare professionals is challenging. A multidisciplinary group videoconferencing approach (MGVA) can be helpful. Our aim was to provide evidence on MGVA's feasibility in managing CNSLBP and its impact on clinical practice.

**Methods:**

We conducted an open-label, randomized, controlled, parallel-group pilot clinical trial with CNSLBP patients irresponsive to conservative treatment. Patients between 18 and 67 years of age were randomly assigned (1:1) to either Standard-of-Care + MGVA (experimental group) or Standard-of-Care alone (control group). MGVA consisted of integrated sessions for physical rehabilitation/physiotherapy, psychology, and social work treatments. The control group received standard clinical practice treatment.

The feasibility was assessed by the number of study procedures completed to at least 80% as planned. The impact on clinical practice was evaluated by the number of patients who changed their status from "candidate" to "non-candidate" to low back surgery as the treatment of choice for CNSLBP. The SF–36, Oswestry Disability Index, and TMMS–24 questionnaires were used. We performed a whole population-based descriptive analysis.

**Results:**

We included 20 patients, but only 18 were randomized (2 withdrew consent before randomization). The mean (SD) age was 53.1 (5.9) years, and mostly women (13/18); 7 were actively employed.

In the experimental group, 6/9 (67%, 95%CI:35–88%) patients attended at least 80% of the scheduled procedures, while in the control group, 8/9 (89%, 95%CI:57–98%) did. Additionally, 1 out of 4 (25%) patients in the experimental group changed their status from "candidate" for low back surgery to "non-candidate". None of the 2 patients in the control group did so. We found differences between groups in the SF-36 mental health component (*p*-value:0.061), Oswestry Disability Index (*p*-value:0.032), and TMMS-24 Repair component (*p*-value:0.014) at the end of the trial favoring MGVA.

**Conclusions:**

The multidisciplinary group videoconferencing approach to managing chronic non-specific low back pain was feasible, suggesting overall beneficial effects on patients’ health and could play a role in changing a patient's status from “candidate” to “non-candidate” for surgery.

**Trial registration:**

NCT05093543 (ClinicalTrials.gov), first registered in 26/10/2021.

**Supplementary Information:**

The online version contains supplementary material available at 10.1186/s12891-023-06763-6.

## Introduction

### Background

Life expectancy has progressively improved over the years, but as chronic conditions become more and more common, quality of life (QoL) often does not keep up [1]. Most chronic conditions do not have a single cause, and psychosocial factors play a role in their negative impact on patients' QoL. An example is chronic non-specific low back pain (CNSLBP) [2].

Low back pain (LBP) is a common condition with a high lifetime prevalence (84%) [3] that becomes even more prevalent with age-related degenerative changes [4, 5]. Disease burden estimates [6] ranked LBP and neck pain as the fourth global leading cause of Disability-Adjusted Life Years in 2015 [1]. Pain is currently defined as an unpleasant sensory and emotional experience associated with, or resembling that associated with, actual or potential tissue damage [7]. Personal and social status and psychological background influence pain perception and may contribute to chronification [8]. Non-specific LBP accounts for 85% of cases [9], and the estimated prevalence of CNSLBP is as high as 23%. Notably, approximately 11 – 12% of the population has some degree of CNSLBP-related disability [3]. Individual baseline characteristics, occupational hazards, and psychosocial issues increase the risk of chronification and have been associated with CNSLBP [4, 10, 11]. The disease's natural history may turn patients into candidates for low back surgery once they become irresponsive to conservative treatment (including rehabilitation and Pain Clinic evaluation and follow-up).

The widespread use of ineffective/not cost-effective therapies [5] and poor adherence to LBP treatment guidelines may put patients at unnecessary risk and increase the disease burden [12]. For instance, spinal arthrodesis is frequently used to treat CNSLBP, but its benefits in non-radicular LBP with non-specific degenerative changes are similar to those of intensive multidisciplinary rehabilitation and only modestly greater than standard conservative treatment [13]. So far, the evidence supporting it is still poor, and many patients who undergo surgical treatment experience persistent pain months or years after it.

A global approach encompassing organic and psychosocial aspects has already been proposed in the 1980s [14], highlighting the importance of assessing the extent of psychosocial factors’ influence on symptoms. Some current clinical guidelines [15, 16] support a non-pharmacological multidisciplinary approach for CNSLBP (*i.e.*, physical activity, multidisciplinary rehabilitation, mindfulness/cognitive-behavioral therapy). The multidisciplinary biopsychosocial approach must be conducted by at least 2 healthcare professionals from different areas since it must have a physical component and at least 1 other component of the biopsychosocial model (*i.e*., psychological, social, and occupational) [17]. Physiotherapy programs combined with psychosocial interventions are recommended for CNSLBP, preferably in group sessions [18, 19]. Group experience itself contains therapeutic phenomena [20] (*e.g.*, altruism, group cohesion, interpersonal learning), and group cohesion improves outcomes in both in- and outpatient settings [21] and should be encouraged.

The south metropolitan region of Barcelona already has the necessary means to implement a multidisciplinary approach, but treatment strategies are still uncoordinated. Integrating different healthcare professionals is challenging and may require extra effort, and busy schedules may be an obstacle for patients to gather for regular group sessions. However, technology can be a game-changer. Our working hypothesis was that a multidisciplinary group videoconferencing approach (MGVA)—incorporating psychology and social work sessions in addition to conventional rehabilitation—in patients with CNSLBP irresponsive to conservative treatment is feasible. Therefore, this clinical trial aimed to provide evidence of MGVA's feasibility in managing CNSLBP and its impact on clinical practice.

## Patients and methods

### Study design

*EN-FORMA* consisted of an open-label, randomized, controlled, parallel-group, pilot clinical trial in patients with CNSLBP irresponsive to conservative treatment. Irresponsiveness was defined as pain persistence severe enough to drive the patient to keep seeking additional help to soothe it.

The study protocol received Institutional Review Board (Bellvitge University Hospital Ethics Committee) approval on April 8^th^, 2021 (PR114/21). This Institutional Review Board acted as the Trial’s coordinating Ethics Committee, and was registered on ClinicalTrials.gov (NCT05093543, first registered in 26/10/2021). All patients included in this trial gave their written informed consent to participate. This clinical trial was conducted in accordance with the Declaration of Helsinki and Good Clinical Practice Guidelines (ICH E6 R2). Patient confidentiality was guaranteed following the current Spanish legislation (LOPD 3/2018). This manuscript complies with the CONSORT 2010 statement.

### Study population and Eligibility criteria

Patients were recruited at the Bellvitge University Hospital (a tertiary hospital), Viladecans Hospital (a secondary hospital), Delta del Llobregat and Santa Eulalia Primary Care Centers, and L’Hospitalet de Llobregat Mental Health Unit.

Inclusion criteria were: (1) patients between 18 and 67 years of age (*i.e.*, working-age population), (2) of both sexes, (3) diagnosed with CNSLBP irresponsive to conservative treatment (including rehabilitation and Pain Clinic evaluation and follow-up), (4) whose predominant symptom was low back pain (and NOT pain radiating to the extremities), and (5) who signed the written informed consent.

Exclusion criteria were: (1) history of previous lumbar arthrodesis; (2) diagnosis of lumbar instability or non-degenerative pathologies (*e.g.*, fractures, tumors, infections) that justify their chronic LBP; (3) inability to move independently; (4) any contraindication to performing light aerobic exercise or physical therapy exercises for LBP; (5) any psychiatric condition that the study team deemed limiting to comply with the study procedures; (6) candidates for low back surgery with a scheduled intervention during the study period; (7) patients with scheduled pain clinic intervention or extra rehabilitation sessions during the study period; (8) patients deemed by the study team as lacking motivation or showing no commitment to the program; (9) patients without access to a device with internet connection and/or webcam (*e.g.*, smartphone, tablet, or computer).

The following anonymized data were gathered into an ad hoc-created electronic case report form (eCRF): baseline and demographic data, past medical history, neuromuscular examination results, and regular use of analgesics.

### Randomization sequence generation

Patients who met all the inclusion criteria and none of the exclusion criteria were included in the study. Participants were randomly assigned following a 1:1 allocation ratio to either the experimental group (Standard of Care [SoC] + MGVA) or control group (SoC) through computer-generated random sequence numbers. The randomization was performed through the eCRF based on the REDCap platform (Research Electronic Data Capture software, REDCap Consortium).

#### Allocation concealment and blinding

Given that this is an open-label study, no concealment mechanisms or blinding procedures were adopted.

#### Implementation

After randomization, each patient received a phone call informing their assigned group; the assigned treatment was once more explained. Patients assigned to the experimental group were emailed a link to access the first group videoconferencing session.

### Study procedures

The study duration was from the screening and randomization visit (day 0) to 12 months after (End of Trial [EoT] visit). Individual face-to-face visits were conducted separately by a physiatrist, psychologist, and a social worker at the beginning and at 6 months. Likewise, all patients were asked to answer the study questionnaires at the beginning and at 6 months (the only exception was the Short-Form 36 [SF-36] questionnaire, which patients were also required to answer at two months —once the MGVA sessions finished).

CNSLBP patients irresponsive to conservative treatment were screened while attending their scheduled outpatient clinic visits with any study team member, regardless of being candidates for low back surgery or not. Patients eligible for surgery primarily had conditions such as discopathy or facet joint arthrosis of varying degrees, causing severe pain irresponsive to conservative treatment.

Subsequently, all patients were reviewed in order from the most recently visited to the latest. The first 20 patients who met all the inclusion criteria and none of the exclusion criteria, and who accepted to participate were selected. Patients were provided full and adequate verbal and written information regarding the objective and procedures of the study and the possible risks involved. Patients were informed that their participation in the trial was voluntary and unpaid and that they could refuse to participate or withdraw from the study at any time without any penalty or loss of benefits to which they were otherwise entitled if not participating.

At the EoT visit (12 months), the patients' eligibility for surgery was reassessed. Those still eligible for surgery continued their standard follow-up with the responsible surgeon. Those who changed their status to "non-candidates" were scheduled for outpatient clinic control visits. Withdrawn patients had all study-related procedures halted and remained only with the medical procedures, tests, and clinical visits scheduled before enrollment.

All study procedures were covered by the Catalan Institute of Health and did not incur in any additional costs neither for the patients nor for the healthcare system.

### Interventions

1. Experimental group

Patients randomized to the experimental group received SoC and MGVA for CNSLBP. MGVA consisted of a biopsychosocial rehabilitation plan that included 8 group videoconferencing sessions of physical rehabilitation/physiotherapy and psychosocial intervention offered as an integrated program. All sessions were scheduled and periodic; patients in this group received a single-use link by email to join the weekly 2-h group videoconference session. The sessions’ content is summarized in Table 1.

**Table 1:** Multidisciplinary approach sessions’ content

**Physical Rehabilitation/Physiotherapy (Total of 8 Sessions)**
∉ Initial health education chat to improve postural hygiene and ergonomics, offer basic anatomical knowledge on pain transmission routes and central sensitization, and explain the program's schedule and objectives ∉ Group exercise program (awareness through movement, lumbar kinesitherapy, and strength and resistance training) ∉ Patients received orientation to keep a regular training regimen at home (both between sessions and after the trial ended)
**Psychology Treatment (Total of 8 Sessions)**
∉ Psychoeducational intervention^a^ (to address psychological factors that could be affecting individual chronic pain perception and to aid transformations in the most active and adaptive coping strategies): ○ 1^st^ session: group members were introduced and the therapists established the operating procedures ○ Sessions 2 – 7: Psychoeducational intervention ○ 8^th^ session: Therapists looked for sings of separation anxiety and mourning due to the end of the experience
**Social Work Treatment (Total of 8 Sessions)**
∉ Based on Antonovsky's Salutogenesis Concept [20] ∉ Social Work group intervention: ○ 1^st^ session: participants were introduced and an initial group assessment was performed ○ Sessions 2 – 7: pain management topics were approached from a social-work perspective ○ 8^th^ session: reflected on the group's objective concepts; identified social resources that could be used; program evaluation; group farewell

^a^Unlike the conventional psychoeducational model, topics were introduced to the extent that an explicit or implicit interest was detected in the participants. We aimed to stimulate a more active attitude linked to the participants' motivation

2) Control group

Patients randomized to the control group received the SoC treatment per usual clinical practice.

SoC after conservative treatment failure consisted of patient referral for follow-up at the discretion of the healthcare providers involved (*e.g.*, physiatrist/physiotherapist, pain clinic specialist, general practitioner, and/or spine surgeon) and considering the patients' preferences on the matter.

### Outcomes

The primary endpoints were clinical trial feasibility and its impact on clinical practice. The clinical trial feasibility was assessed by the number of study procedures completed to at least 80% as planned. In the experimental group, we assessed (i) the number (percentage) of scheduled visits attended by the included patients; (ii) the number (percentage) of questionnaires fulfilled by the included patients; (iii) the number (percentage) of scheduled MGVA sessions attended by the included patients; and (iv) the number (percentage) of MGVA sessions performed as planned was also assessed (sessions were considered satisfactory when performed coordinately by the three therapists, when no internet connection issues occurred, when they started and finished on the expected time and date, and when patients had a proactive role). In the control group, we assessed (i) the number (percentage) of scheduled visits attended by the patients; and (ii) the number (percentage) of questionnaires fulfilled by them.

The impact on clinical practice was evaluated by the number of patients who changed their status from “candidate” to “non-candidate”/ “non-candidate” to “candidate” for low back surgery as the treatment of choice for CNSLBP.

Secondary endpoints were:

1. QoL evaluated by the SF-36 questionnaire [22];
2. Low back pain disability estimated by the Oswestry Disability Index (ODI) score [23, 24];
3. Physical Activity assessed by the International Physical Activity Questionnaire – Short Form (IPAQ-SF) score [25, 26];
4. Anxiety and Depression evaluated by the Hospital Anxiety-Depression Scale (HADS) score [27–29];
5. Chronic pain coping strategies assessed by the Vanderbilt Pain Management Inventory (VPMI) score [30–32];
6. Awareness and emotional self-regulation evaluated by the Trait Meta-Mood Scale (TMMS-24) score [33, 34];
7. Mental wellbeing evaluated by the Warwick-Edinburgh Mental Wellbeing Scale (WEMWBS) score [35];
8. Level of social support estimated by the Oslo-3 questionnaire score [36, 37].

We used the Spanish version of all the above-mentioned scales and questionnaires. All randomized patients received the questionnaires by email at the beginning and 6 months after the study start. The SF-36 questionnaire was also sent after concluding the last MGVA session (two months after the beginning of the study). All answers were automatically inserted in the eCRF.

### Statistical analysis

Given the exploratory nature of this pilot clinical trial and the absence of previous studies comparing MGVA with the conventional conservative treatment, we did not perform a formal sample size calculation. Therefore, we set a target of 20 patients as a reasonable and affordable goal for this pilot study.

All gathered data was summarized by study groups using appropriate statistical methods according to their type. Continuous variables were presented with mean and standard deviation, except for those with asymmetry or lack of normality which were described with median and interquartile range. Categorical variables were reported as the number of cases and the percentage of the total. The demographic and clinical profiles of the included patients were described in total, and by study group. The secondary endpoints were analyzed following the same procedure described below for the QoL. The evolution of the average QoL throughout the 6 months period was described with graphs and descriptive statistics. QoL improvement between the study groups was calculated through a covariance analysis, considering QoL at 2 months (measured by the SF-36 questionnaire) as the dependent variable as well as the study group and QoL at the baseline visit (also measured by the SF-36 questionnaire) as independent variables. Improvement was quantified by estimating the expected marginal effect on QoL at 2 months by study group.

A 95% confidence interval (95% CI) for the estimations was provided whenever possible. The statistical analyses were performed with the R program version 4.1.2 for Windows® (R Foundation for Statistical Computing).

No interim analysis and no subgroup or adjusted analyses were performed. Also, this study did not have a Data Safety and Monitoring Committee; monitoring was conducted by study team members not involved in patient inclusion and follow-up.

## Results

### Baseline characteristics

**Fig. 1:**
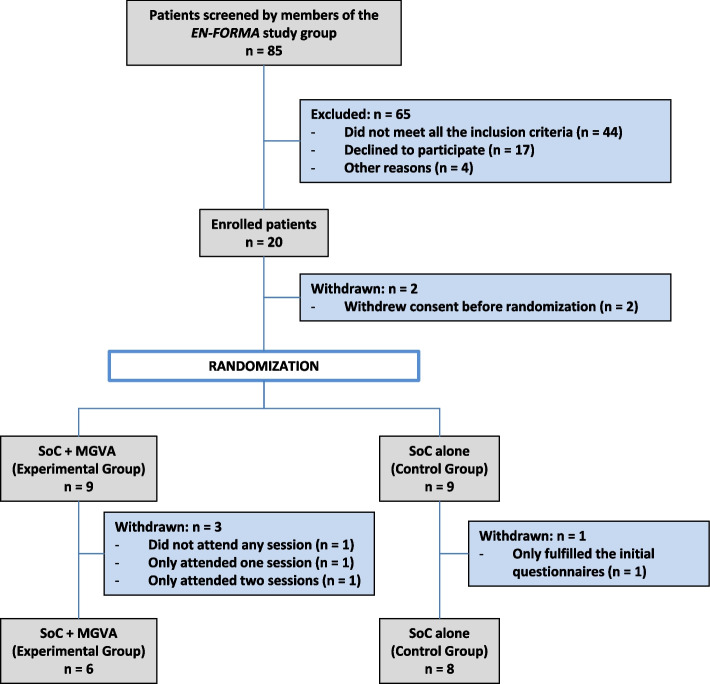
Study flowchart. MGVA: Multidisciplinary Group Videoconferencing Approach; SoC: Standard of Care

A total of 20 patients with CNSLBP irresponsive to conservative treatment were included in the study in April 2021. The study duration from the screening and randomization visit (day 0) to the EoT visit was 12 months. Two patients withdrew consent prior to randomization. The remaining 18 patients were randomized to either the SoC + MGVA (experimental group) or SoC (control group) (see Fig. 1). The overall dropout rate after randomization was 22% (4/18 patients): in the experimental group, 3/9 (33.3%) patients dropped-out from the study due to busy schedules or personal reasons; in the control group, 1/9 (11.1%) patient were withdrawn for refusing to answer the study questionnaires. Six patients (4 in the experimental group and 2 in the control group) were considered candidates for low back surgery as the treatment of choice for CNSLBP by the responsible surgeon before starting any study procedure.

Table 2 shows the demographic and baseline characteristics of all randomized patients. The demographic and baseline characteristics of those patients who finished the study (after withdrawals) were similar to the randomized population, except for the employment status (experimental group: 2 [33.3%] active patients; control group: 5 [62.5%] patients). The demographic and baseline characteristics of those patients who finished the study (after withdrawals) is available in the Supplementary material 1.

**Table 2:** Demographic and baseline characteristics of the randomized study population

	**Experimental (SoC + MGVA)**	**Control (SoC alone)**
	*N* = *9*	*N* = *9*
Age, Mean (SD)	51.1 (4.81)	55.2 (6.40)
Sex, N (%):		
Men	1 (11.1%)	4 (44.4%)
Women	8 (88.9%)	5 (55.6%)
BMI (Kg/m^2^), Mean (SD)	26.4 (5.29)	26.6 (3.74)
**Harmful habits**		
Tobacco smokers, N (%):		
No	3 (33.3%)	8 (88.9%)
Yes	6 (66.7%)	1 (11.1%)
Alcohol consumption, N (%):		
No	8 (88.9%)	7 (77.8%)
Yes	1 (1.11%)	2 (2.22%)
**Employment status, N (%):**		
Active	4 (44.4%)	5 (55.6%)

*SD* Standard Deviation, *BMI* Body Mass Index

### Primary endpoints

#### Clinical trial feasibility

**Fig. 2:**
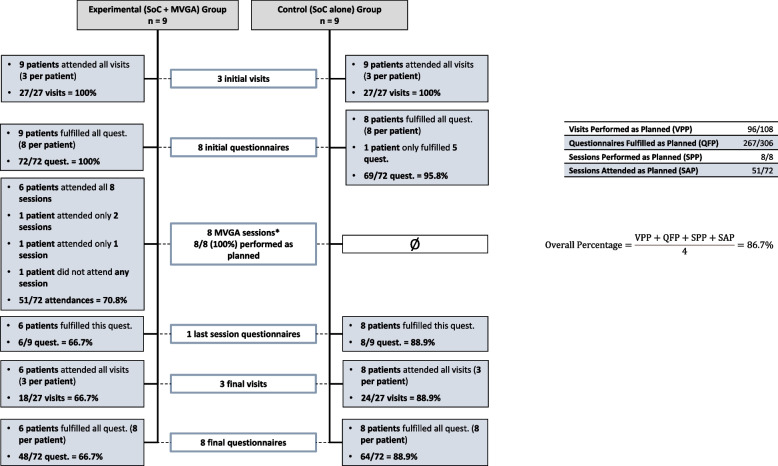
Feasibility measured by percentage of events that occurred as planned. ^*^MGVA sessions were performed coordinately by the three therapists. Quest.: questionnaires

This analysis was performed in the randomized population. The number of patients who completed at least 80% of the scheduled study procedures was 6 out of 9 (67%, 95% CI: 35 – 88%) in the experimental group and 8 out of 9 (89%, 95% CI: 57 – 98%) in the control group. Figure 2 depicts in detail the visits and sessions performed along the study.

#### Impact on clinical practice

This analysis was performed at the EoT (12 months). Of the 6 patients candidate for low back surgery as the treatment of choice for CNSLBP at baseline, 1 out of 4 (25%) patients in the experimental group and 0 out of 2 (0%) patients in the control group changed from “candidate” to “non-candidate”. All 8 “non-candidate” patients at baseline remained as “non-candidates” at the EoT in both groups.

### Secondary outcomes

Secondary outcomes were assessed in those patients who completed the study: 6 patients in the experimental group and 8 patients in the control group.

#### Quality of life

Evolutionary improvements in mental health (*p*-value: 0.061) favor the MGVA treatment over the SoC.

**Fig. 3:**
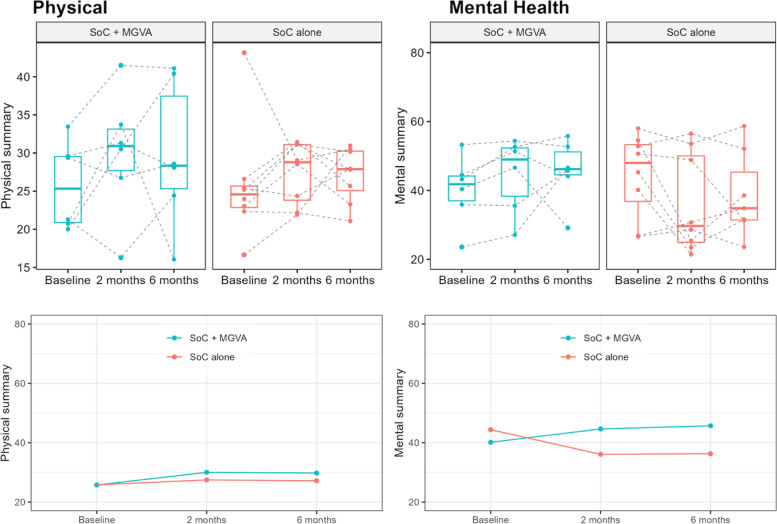
QoL evaluated by the SF-36 questionnaire

Notably, patients in the experimental group considerably improved their mean SF-36 mental health score, while those in the SoC worsened it. Figure 3 shows the evolution of the QoL scores based on the SF-36 questionnaire.

#### Low back pain disability

**Fig. 4:**
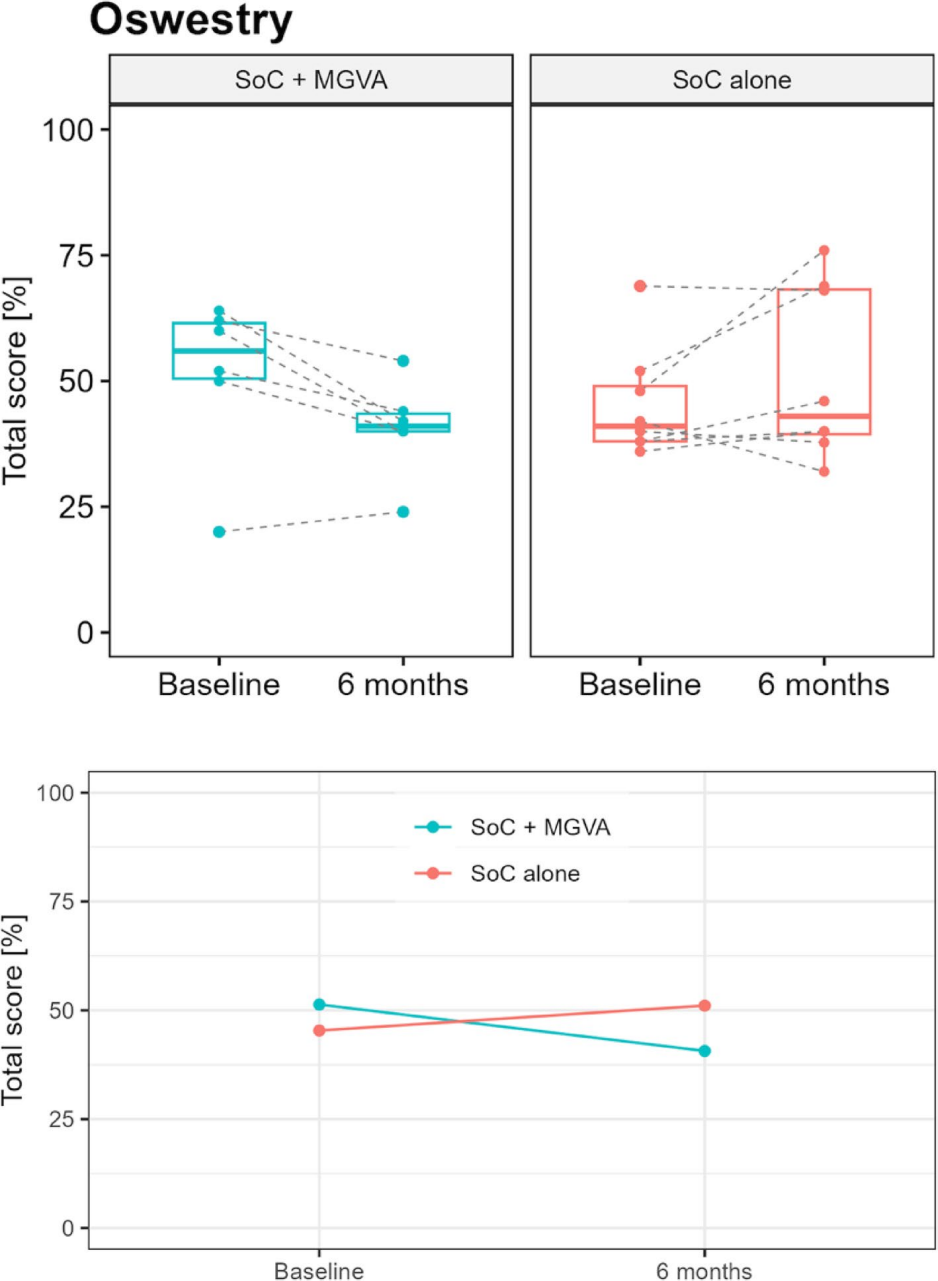
Oswestry Disability Index

Five out of 6 (83%) patients in the experimental group and 3 out of 8 (38%) in the control group improved their ODI score (*p*-value: 0.032), but 2 of these 3 patients in the control group only improved their scores by 1 and 2 points. Figure 4 shows the patients’ Oswestry Disability Index evolution.

ODI scores provide a classification of a patient’s disability level: minimal disability (0 – 20%), moderate disability (21 – 40%), severe disability (41 – 60%), crippled (61—80%), and bed-bound (81 – 100%). Overall, more patients in the experimental group changed to a lesser disability category than in the control group (see Supplementary material 2).

#### Physical activity

At the beginning of the study, 5 out of 6 (83%) patients in the experimental group and all (100%, 8/8) in the control group engaged in regular walking activity. After 6 months, all (6/6, 100%) patients in the experimental group were able to walk regularly; however, in the control group, the number of patients walking regularly decreased to 6 out of 8 (75%). Notably, no patients in the experimental group were capable to tolerate moderate physical activity at baseline, but 1 (17%) patient in this group tolerated it at the EoT. IPAQ results are shown in the Supplementary material 3.

#### Anxiety and depression

**Fig. 5:**
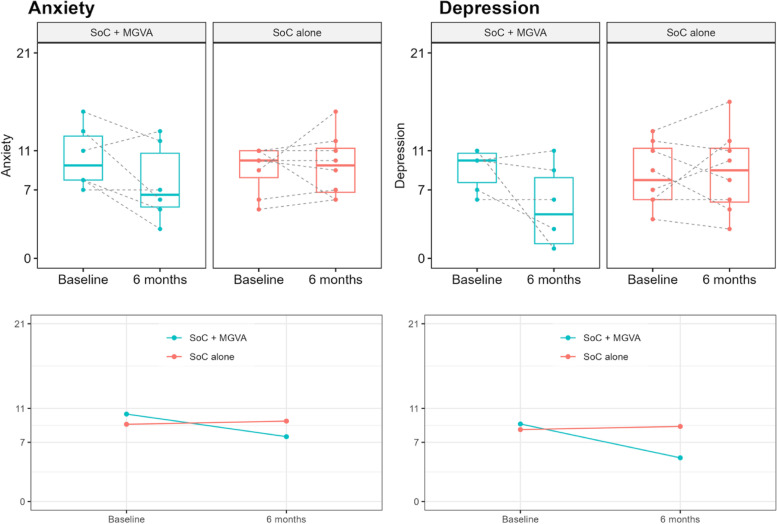
Hospital Anxiety and Depression Scale (HADS)

HADS has two subscales: Anxiety and Depression. Patients are classified as normal (score: 0 – 7), borderline (score: 8 – 10), or abnormal (score: 11 – 21) in each subscale, depending on their scores. More patients in the experimental group reached the “normal” category at 6 months than in the control group (higher absolute and relative frequencies). Likewise, the depression subscale showed a trend towards reduction in the experimental group after MGVA (*p*-value: 0.061) and, overall, more patients in this group improved their category (see Fig. 5 and Supplementary material 4).

#### Chronic pain coping strategies

Similar results were found between groups regarding chronic pain coping strategies (evaluated by the VPMI) (*p*-value: 0.207). Four (67%) patients in the experimental group adopted fewer passive pain coping strategies, while 3 (50%) patients used more active strategies. In the control group, 3 (38%) patients adopted fewer passive strategies, while 3 (38%) patients adopted more active strategies. However, there was a trend in the experimental group to decrease its mean VPMI passive score while increasing its active counterpart. In contrast, in the control group we observed a trend to increase the mean passive score while decreasing its mean active counterpart. Supplementary material 5 shows the boxplot and mean scores evolution of the VPMI (chronic pain coping strategies).

#### Awareness and emotional self-regulation

We used the TMMS-24 to assess awareness and emotional self-regulation. In the attention component, 3 (50%) patients improved their scores in the experimental group, but only 1 (17%) patient improved the attention status from overattentive to normal. In the control group, 4 (50%) patients improved their scores, but no change in status was observed. Regarding the clarity component, 3 (50%) patients in the experimental group and 1 (13%) patient in the control group improved their status.

On the other hand, 3 (50%) patients in the experimental group and 4 (50%) in the control group improved their TMMS-24 Repair component. Two (33%) patients in the experimental group upgraded their status and 1 (13%) patient in the control group downgraded his/hers. Improvements in the experimental group compared to the control group were found in the TMMS-24 Repair component (*p*-value:0.014) at 6 months. Supplementary Material 6 shows the boxplot and mean TMMS-24 scores at baseline and 6 months.

#### Mental wellbeing

Mental wellbeing showed a trend towards better scores in the experimental group after MGVA (*p*-value: 0.199), evaluated by the WEMWBS score. Four (50%) patients in the experimental group improved their WEMWBS scores, while 6 (75%) in the control group worsened their scores and the 2 remaining patients’ results are missing. Supplementary material 7 shows the boxplot and mean WEMWBS scores at baseline and 6 months.

#### Level of estimated social support

Similar results were found between groups in the level of estimated social support assessed by the Oslo-3 scale (*p*-value: 0.692). Two (33%) patients in the experimental group and 4 (50%) in the control group improved their Oslo-3 scores. Supplementary material 8 shows the boxplot and mean Oslo-3 scores.

### Adverse events

No adverse events related to the study treatments were gathered.

## Discussion

To the best of our knowledge, this is the first pilot randomized clinical trial using videoconferencing as a tool to coordinate and perform multidisciplinary sessions for CNSLBP. Other authors had previously studied the multidisciplinary approach for CNSLBP but conducted face-to-face. Our results show that multidisciplinary group videoconferencing approach is feasible within our setting to coordinate different healthcare professionals and provide the biopsychosocial model to the CNSLBP patients herein. Overall, multidisciplinary group videoconferencing approach seemed to have a positive effect, improving almost all the evaluations performed: QoL, low back pain disability, physical activity, anxiety and depression, awareness and emotional self-regulation, mental wellbeing, and especially in patients’ mental health and disability. Besides, multidisciplinary group videoconferencing approach could play a role in changing a patient's status from “candidate” to “non-candidate” for low back surgery. Videoconferencing may be a way of bypassing busy schedules, distances, and urban mobility issues, improving treatment adherence and reducing healthcare costs.

### Multidisciplinary approach

A 2022 systematic review on the psychological interventions for CNSLBP reported that the most sustainable effects for physical function and fear avoidance were achieved with pain education programs and for pain intensity was behavioral therapy, both delivered with physiotherapy care [38]. A randomized controlled trial reported that a multidisciplinary program of task-oriented exercises integrated with cognitive behavioral therapy was superior to group-based traditional exercises in reducing disability, kinesiophobia, catastrophizing, and enhancing the QoL of chronic LBP patients [39]; a subsequent systematic review found similar results between mindfulness-based stress reduction and cognitive-behavioral therapy chronic pain management [40].

Despite the currently available evidence supporting the multidisciplinary approach for CNSLBP, it is not widely implemented as part of the usual clinical practice. This might be because of the challenges in coordinating different healthcare professionals and the many patient-referral routes (*e.g.*, primary care and rehabilitation centers, pain clinics, and tertiary hospitals). Our results showed that a high percentage of the study procedures occurred as planned.

### Group videoconferencing

Using group videoconferencing to manage chronic conditions has already been attempted before, and a quick PubMed® search generates results dating back over ten years ago. A 2012 videoconferencing management protocol by the San Francisco Veteran Affairs Medical Center integrates cognitive-behavioral therapy and physical therapy [41].

A pre-SARS-CoV-2-pandemic systematic review found home-based groups by videoconferencing feasible even for those with limited digital literacy [42]. Outcomes were similar to in-person groups, and it may be a potential alternative to overcome accessibility barriers (*e.g.*, limited mobility, socially isolated patients, those who fear meeting new people face-to-face, and those living in rural areas) [42]. Subsequent meta-analyses pointed in the same direction: the combined use of mobile devices and usual care interventions was found to be superior to usual care alone in reducing pain intensity and disability in LBP patients [43], whereas telerehabilitation in physical therapy for osteoarthritis, LBP, and hip and knee replacement had similar results to in-person rehabilitation or better than no rehabilitation [44]. A recent clinical trial found that integrated cognitive-behavioral therapy delivered by videoconferencing may be cost-effective and reduce pain interference [45].

Furthermore, recent studies examining the effectiveness of video exercise-based telerehabilitation in patients with chronic low back pain [46] and the viability of telerehabilitation as an option during the COVID-19 pandemic [47] have reported positive clinical outcomes, satisfaction, and motivation, further supporting the potential benefits of incorporating telerehabilitation into chronic condition management.

It is not farfetched to expect a better experience (for both patients and healthcare professionals) through videoconferencing than with other devices that do not allow capturing facial expressions and body language in real time. It is still unclear whether MGVA would influence patients’ clinical responses. However, it would improve accessibility and essentially provide them with basic treatment tools and professional supervision, although in an unconventional way. Importantly, we acknowledge that the adherence to all study-related procedures within the experimental group was lower than in the control group; however, the clinical impact on experimental group patients was still favorable. Thus, it is reasonable to think that higher adherence could lead to potentially better outcomes. This remains a significant challenge for studies yet to come.

### Avoiding surgery

Despite the growing body of evidence that supports combining exercise therapy and psychosocial interventions for managing CNSLBP, many patients still undergo spinal fusion surgery instead of joining a multidisciplinary program. Guidelines recommend the cautious use of medication, imaging, and surgery. Still, gaps between evidence and practice exist, with limited use of recommended first-line treatments and inappropriate overuse of imaging, rest, opioids, spinal injections, and surgery [12]. As previously mentioned, spine surgery for CNSLBP has similar benefits to intensive multidisciplinary rehabilitation [13]. MGVA seemed to positively impact our clinical practice by influencing the decision of ruling out the surgical treatment in 1 out of 4 patients that had been considered eligible for low back surgery in the experimental group. Multidisciplinary approaches may improve the QoL of CNSLBP patients without the burdens caused by spinal surgeries, both to patients and the healthcare budget.

Additionally, multidisciplinary pain interventions have been reported more cost-effective than non-multidisciplinary ones, in terms of Quality-Adjusted Life Years [48]. Other authors reported that multidisciplinary pain interventions were also superior to physical treatment alone but only found it cost-effective in those irresponsive to conventional treatment [17].

Overcoming the logistical issues when coordinating a multidisciplinary approach, changing patients’ mindset to “treating themselves” instead of “being treated”, and dodging the prosthetic industry pressure are challenging but achievable steps to implement this program. In our study, 1/4 (25%) patients offered a surgical approach utterly refused it after the study ended. Despite our small sample size, we expect the continuing implementation of multidisciplinary programs to reduce the number of spinal surgeries performed. However, larger clinical trials are needed to support this expectation.

### Limitations and generalizability

This is a pilot clinical trial with a small sample size and carried out in a specific geographic area, which could lead us to under- or overestimate the generalizability of the results. Additionally, this was the first time our therapists carried out a multidisciplinary group videoconferencing approach (even though they all have years of expertise in their fields). The therapists’ expertise can influence on results obtained [49].

The open-label design could also play a limiting role. Patients’ awareness of the randomized treatment received might have influenced their expectations and their subjective responses. The psychological sensitivity and predisposition of participants in the experimental group might have differed from those in the control group, as the former involved trying a new treatment after previous unsuccessful attempts. Consequently, the predisposition to improve in the experimental group may have been greater. Also, unblinded therapists/assessors may raise concerns of bias; however, we used objective tools to assess the study endpoints to keep observer bias to a minimum, and the assessment of candidacy for surgery was performed by an independent surgeon (not a study team member).

## Conclusion

Our results suggest that, within our setting, multidisciplinary group videoconferencing approach is feasible for coordinating different healthcare professionals and providing the biopsychosocial model to the CNSLBP patients herein. Although our pilot study has shown overall beneficial effects, improving different dimensions such as QoL, low back pain disability, physical activity, anxiety and depression, and awareness and emotional self-regulatioN we acknowledge they should be interpreted with caution, given the small sample size and uneven allocation of surgical candidates between groups. Notwithstanding, the multidisciplinary group videoconferencing approach seems promising in reducing spinal surgeries, their associated complications, and the associated healthcare costs. Further clinical trials are warranted to validate these preliminary findings.

### Supplementary Information

**Additional file 1: Supplementary Material 1.** Demographic and Baseline Characteristics of Patients who Finished the study (after withdrawals).

**Additional file 2: Supplementary Material 2.** Number of Patients in each Oswestry Disability Index Category.

**Additional file 3: Supplementary Material 3.** Level of Regular Activity evaluated by the IPAQ.

**Additional file 4: Supplementary Material 4.** Number of Patients by Category in Each HADS subscale.

**Additional file 5: Supplementary Material 5.** Chronic Pain Coping Strategies evaluated by the VPMI.

**Additional file 6: Supplementary Material 6.** Awareness and Emotional Self-Regulation evaluated by the TMMS-24.

**Additional file 7: Supplementary Material 7.** Mental wellbeing evaluated by the WEMWBS.

**Additional file 8: Supplementary Material 8.** Level of estimated social support evaluated by the Oslo-3 scale.

**Additional file 9: Supplementary Material 9.** Model patient information sheet and other related documentation given to participants and authorized surrogates.

**Additional file 10: Supplementary Material 10.** Model consent form and other related documentation given to participants and authorized surrogates.

## Data Availability

The datasets used and/or analyzed during the current study are available from the corresponding author on reasonable request.
