# Supplementary material for: Feasibility of a multidisciplinary group videoconferencing approach for chronic low back pain: a randomized, open-label, controlled, pilot clinical trial (EN-FORMA)
Source: BMC Musculoskelet Disord. 2023 Aug 9;24:642. doi: 10.1186/s12891-023-06763-6 (PMC10410913; doi:10.1186/s12891-023-06763-6)
Supplement: Supplementary file 1 — Additional file 1: Supplementary Material 1. Demographic and Baseline Characteristics of Patients who Finished the study (after withdrawals). [file 12891_2023_6763_MOESM1_ESM.docx]

**Supplementary Material 1:** Demographic and Baseline Characteristics of Patients who Finished the study (after withdrawals)

|  | **Experimental (SoC + MGVA)** | **Control (SoC alone)** |
| --- | --- | --- |
|  | *N = 6* | *N = 8* |
| Age, Mean (SD) | 52.7 (3.38) | 54.9 (6.78) |
| Sex, N (%): |  |  |
| Men | 1 (16.7%) | 4 (50.0%) |
| Women | 5 (83.3%) | 4 (50.0%) |
| BMI (Kg/m^2^), Mean (SD) | 27.9 (5.49) | 26.2 (3.81) |
| **Harmful Habits** |  |  |
| Tobacco smokers, N (%): |  |  |
| No | 2 (33.3%) | 7 (87.5%) |
| Yes | 4 (66.7%) | 1 (12.5%) |
| Alcohol Consumption, N (%): |  |  |
| No | 5 (83.3%) | 6 (75.0%) |
| Yes | 1 (16.7%) | 2 (25.0%) |
| **Employment status, N (%):** |  |  |
| Active | 2 (33.3%) | 5 (62.5%) |

**SD**: Standard Deviation; **BMI**: Body Mass Index.
