# Supplementary material for: Feasibility of a multidisciplinary group videoconferencing approach for chronic low back pain: a randomized, open-label, controlled, pilot clinical trial (EN-FORMA)
Source: BMC Musculoskelet Disord. 2023 Aug 9;24:642. doi: 10.1186/s12891-023-06763-6 (PMC10410913; doi:10.1186/s12891-023-06763-6)
Supplement: Supplementary file 2 — Additional file 2: Supplementary Material 2. Number of Patients in each Oswestry Disability Index Category. [file 12891_2023_6763_MOESM2_ESM.docx]

**Supplementary Material 2:** Number of Patients in each Oswestry Disability Index Category

**
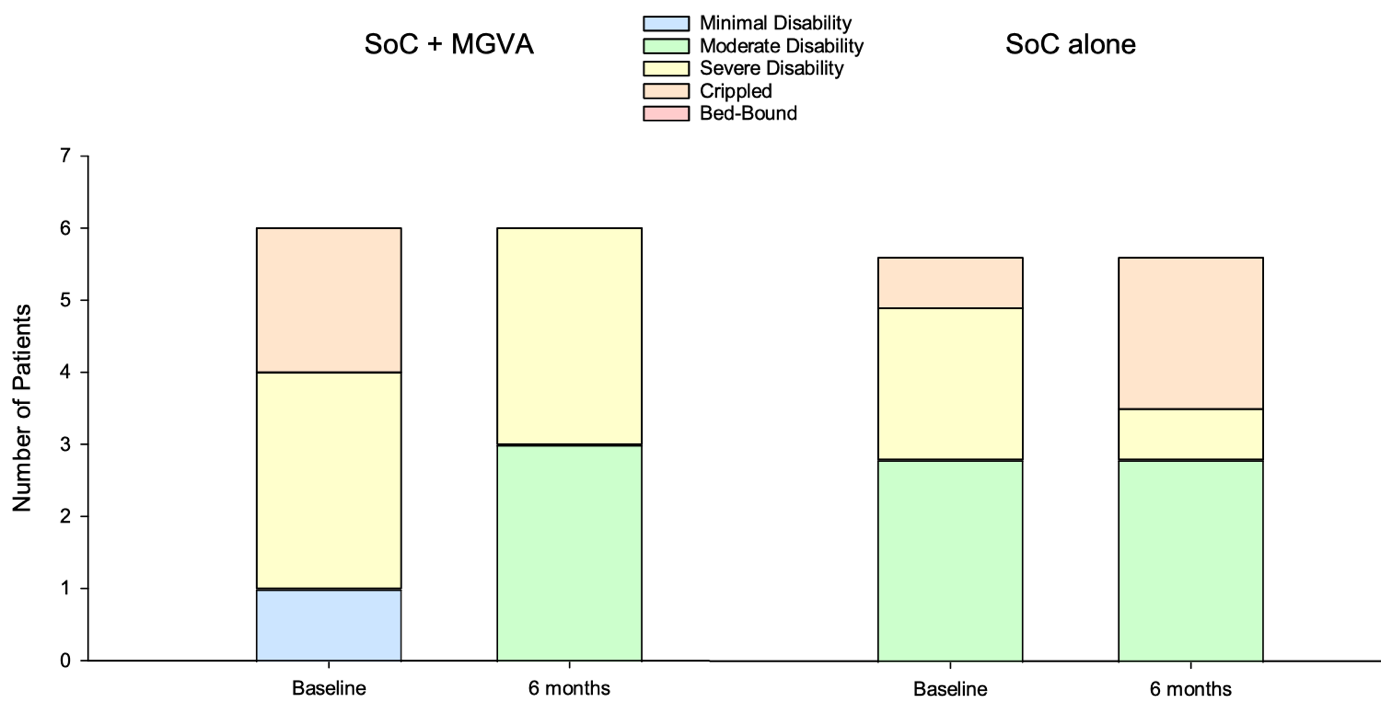
**

**Note:** Categories not displayed in the plot have zero patients. SoC: Standard of Care; MGVA: Multidisciplinary Group Videoconferencing Approach
