# Supplementary material for: Feasibility of a multidisciplinary group videoconferencing approach for chronic low back pain: a randomized, open-label, controlled, pilot clinical trial (EN-FORMA)
Source: BMC Musculoskelet Disord. 2023 Aug 9;24:642. doi: 10.1186/s12891-023-06763-6 (PMC10410913; doi:10.1186/s12891-023-06763-6)
Supplement: Supplementary file 3 — Additional file 3: Supplementary Material 3. Level of Regular Activity evaluated by the IPAQ. [file 12891_2023_6763_MOESM3_ESM.docx]

**Supplementary Material 3:** Level of regular activity evaluated by the IPAQ.

|  | **Baseline** | | **6 months** | |
| --- | --- | --- | --- | --- |
|  | **Experimental (SoC + MGVA)** | **Control (SoC alone)** | **Experimental (SoC + MGVA)** | **Control (SoC alone)** |
| Intense activity | 0 (0%) | 1 (13%) | 0 (0%) | 1 (13%) |
| Moderate activity | 0 (0%) | 1 (13%) | 1 (17%) | 1 (13%) |
| Walking | 5 (83%) | 8 (100%) | 6 (100%) | 6 (75%) |

**SoC:** Standard of Care; **SD**: Standard Deviation; **Moderate activity**: activities that take moderate physical effort and make the patient breathe somewhat harder than normal; **Intense activity**: activities that take hard physical effort and make the patient breathe much harder than normal.
