# Supplementary material for: Feasibility of a multidisciplinary group videoconferencing approach for chronic low back pain: a randomized, open-label, controlled, pilot clinical trial (EN-FORMA)
Source: BMC Musculoskelet Disord. 2023 Aug 9;24:642. doi: 10.1186/s12891-023-06763-6 (PMC10410913; doi:10.1186/s12891-023-06763-6)
Supplement: Supplementary file 4 — Additional file 4: Supplementary Material 4. Number of Patients by Category in Each HADS subscale. [file 12891_2023_6763_MOESM4_ESM.docx]

**Supplementary Material 4:** Number of Patients by Category in Each HADS subscale.

**
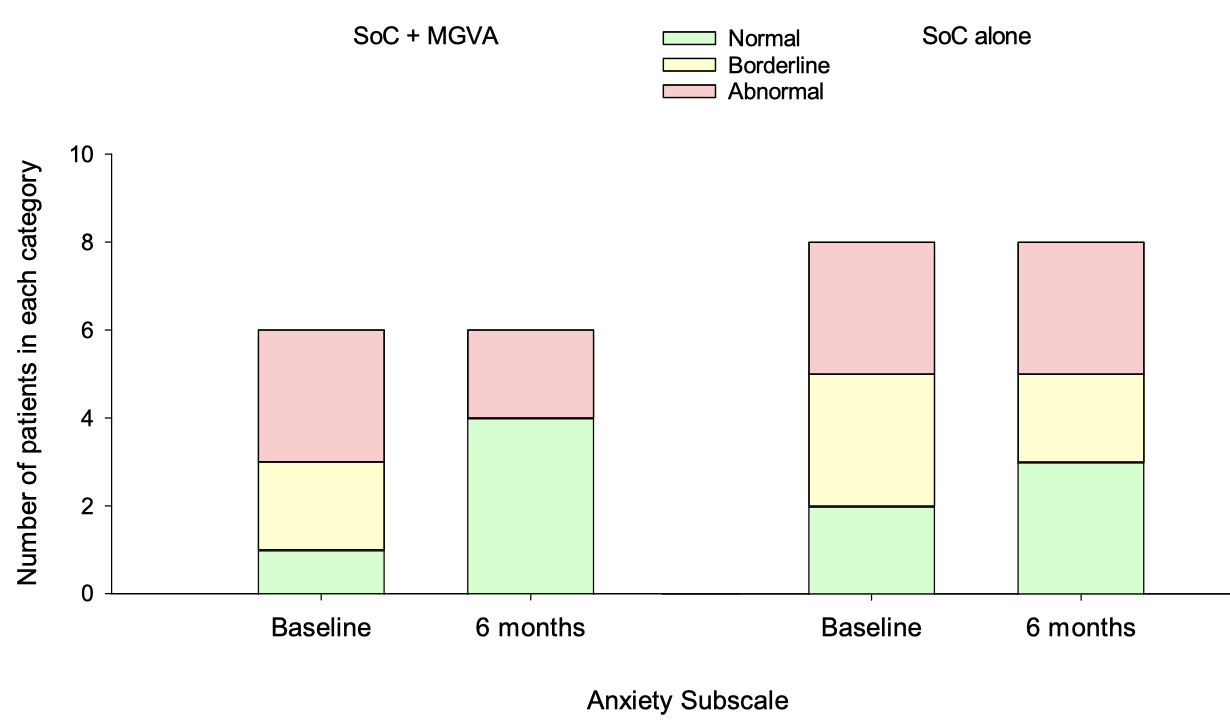
**

**
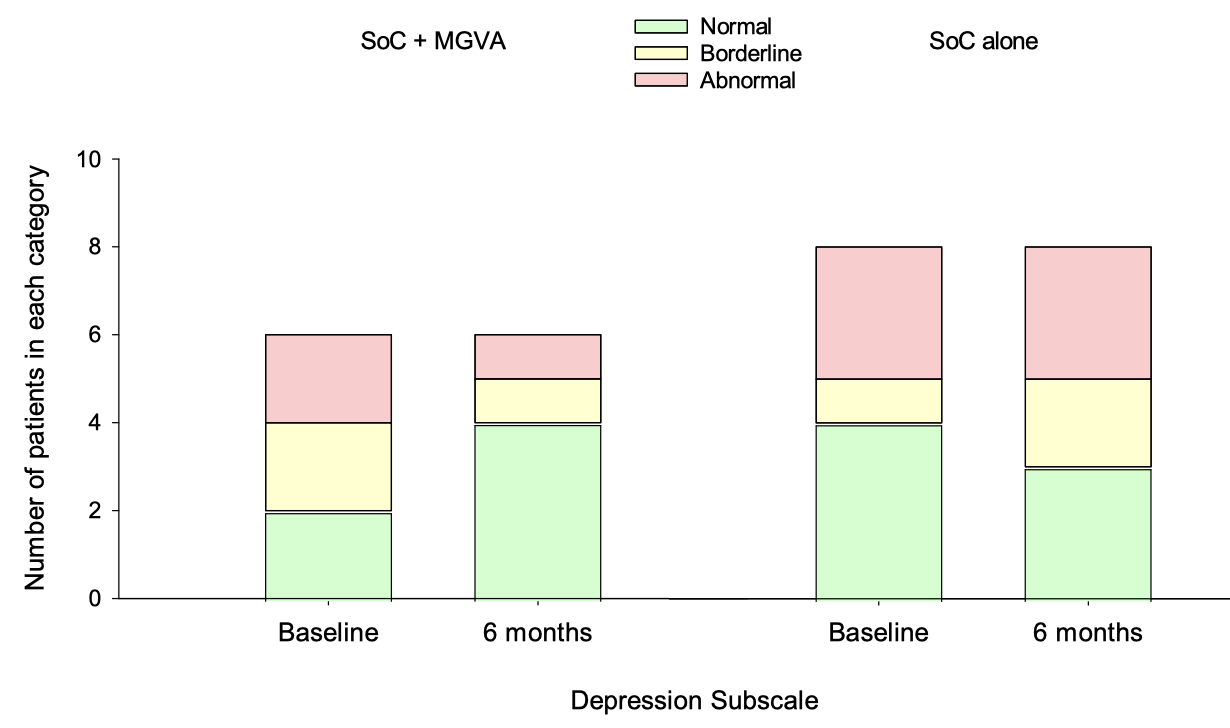
**

**SoC**: Standard of Care; **MGVA**: Multidisciplinary Group Videoconferencing Approach
