# Supplementary material for: Feasibility of a multidisciplinary group videoconferencing approach for chronic low back pain: a randomized, open-label, controlled, pilot clinical trial (EN-FORMA)
Source: BMC Musculoskelet Disord. 2023 Aug 9;24:642. doi: 10.1186/s12891-023-06763-6 (PMC10410913; doi:10.1186/s12891-023-06763-6)
Supplement: Supplementary file 5 — Additional file 5: Supplementary Material 5. Chronic Pain Coping Strategies evaluated by the VPMI. [file 12891_2023_6763_MOESM5_ESM.docx]

**Supplementary Material 5:** Chronic Pain Coping Strategies evaluated by the VPMI

|  | **Baseline** | | **6 months** | |
| --- | --- | --- | --- | --- |
|  | **Experimental (SoC + MGVA)** | **Control (SoC alone)** | **Experimental (SoC + MGVA)** | **Control (SoC alone)** |
| Passive, Mean (SD) | 10.7 (2.58) | 7.88 (2.64) | 8.83 (1.33) | 8.50 (1.85) |
| Active, Mean (SD) | 10.0 (2.19) | 9.75 (1.75) | 11.7 (2.34) | 10.0 (2.14) |

**SoC:** Standard of Care; **SD**: Standard Deviation.

**
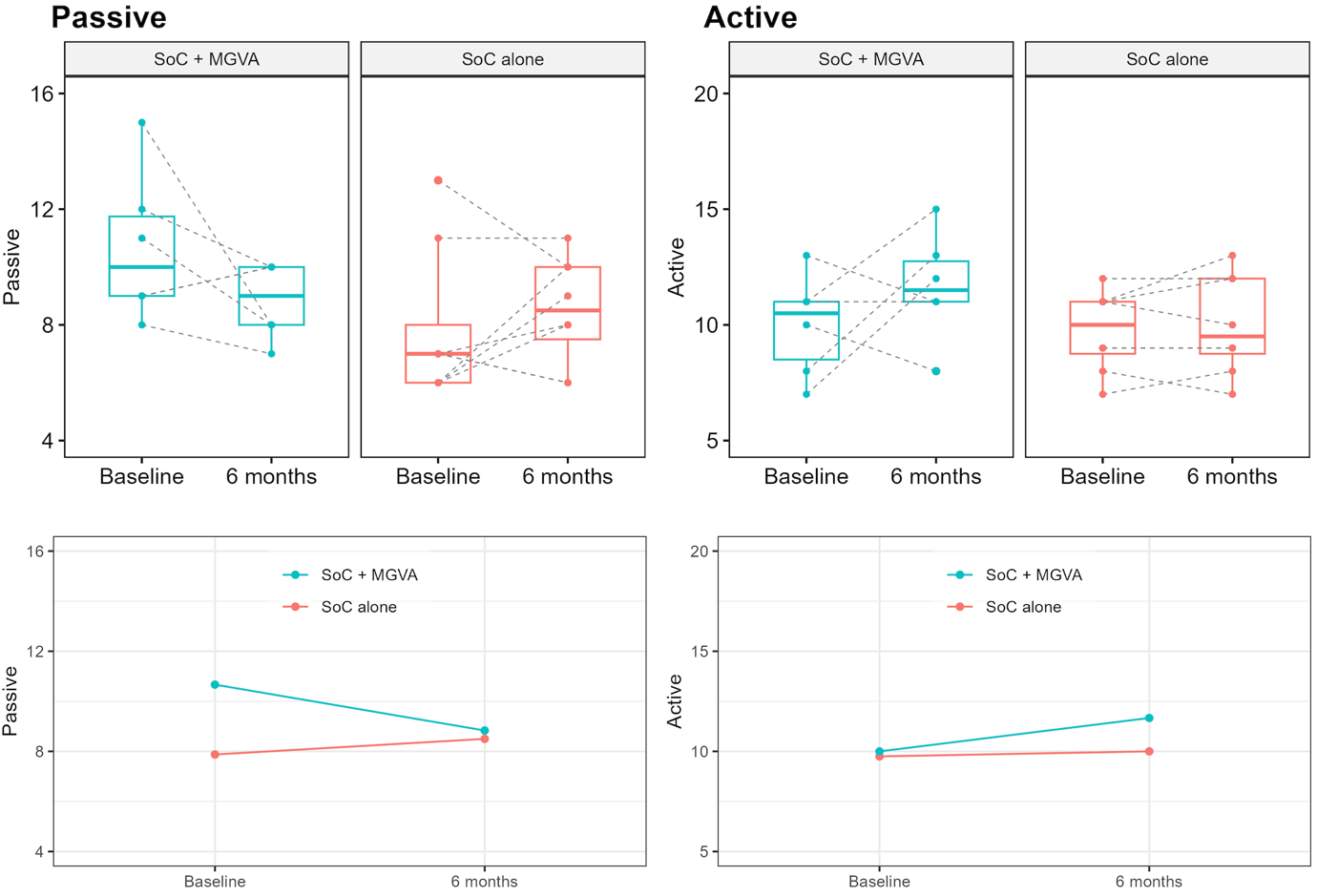
**
