# Supplementary material for: Feasibility of a multidisciplinary group videoconferencing approach for chronic low back pain: a randomized, open-label, controlled, pilot clinical trial (EN-FORMA)
Source: BMC Musculoskelet Disord. 2023 Aug 9;24:642. doi: 10.1186/s12891-023-06763-6 (PMC10410913; doi:10.1186/s12891-023-06763-6)
Supplement: Supplementary file 6 — Additional file 6: Supplementary Material 6. Awareness and Emotional Self-Regulation evaluated by the TMMS-24. [file 12891_2023_6763_MOESM6_ESM.docx]

**Supplementary Material 6:** Awareness and Emotional Self-Regulation evaluated by the TMMS-24

|  | **Baseline** | | **6 months** | |
| --- | --- | --- | --- | --- |
|  | **Experimental (SoC + MGVA)** | **Control (SoC alone)** | **Experimental (SoC + MGVA)** | **Control (SoC alone)** |
| Attention, Mean (SD) | 26.3 (6.95) | 21.9 (9.93) | 24.8 (9.87) | 22.1 (8.68) |
| Clarity, Mean (SD) | 26.8 (7.19) | 22.8 (7.61) | 26.3 (7.53) | 19.2 (7.05) |
| Repair, Mean (SD) | 27.3 (8.59) | 21.4 (7.21) | 31.2 (6.62) | 20.9 (4.97) |

**SoC:** Standard of Care; **SD**: Standard Deviation.

**
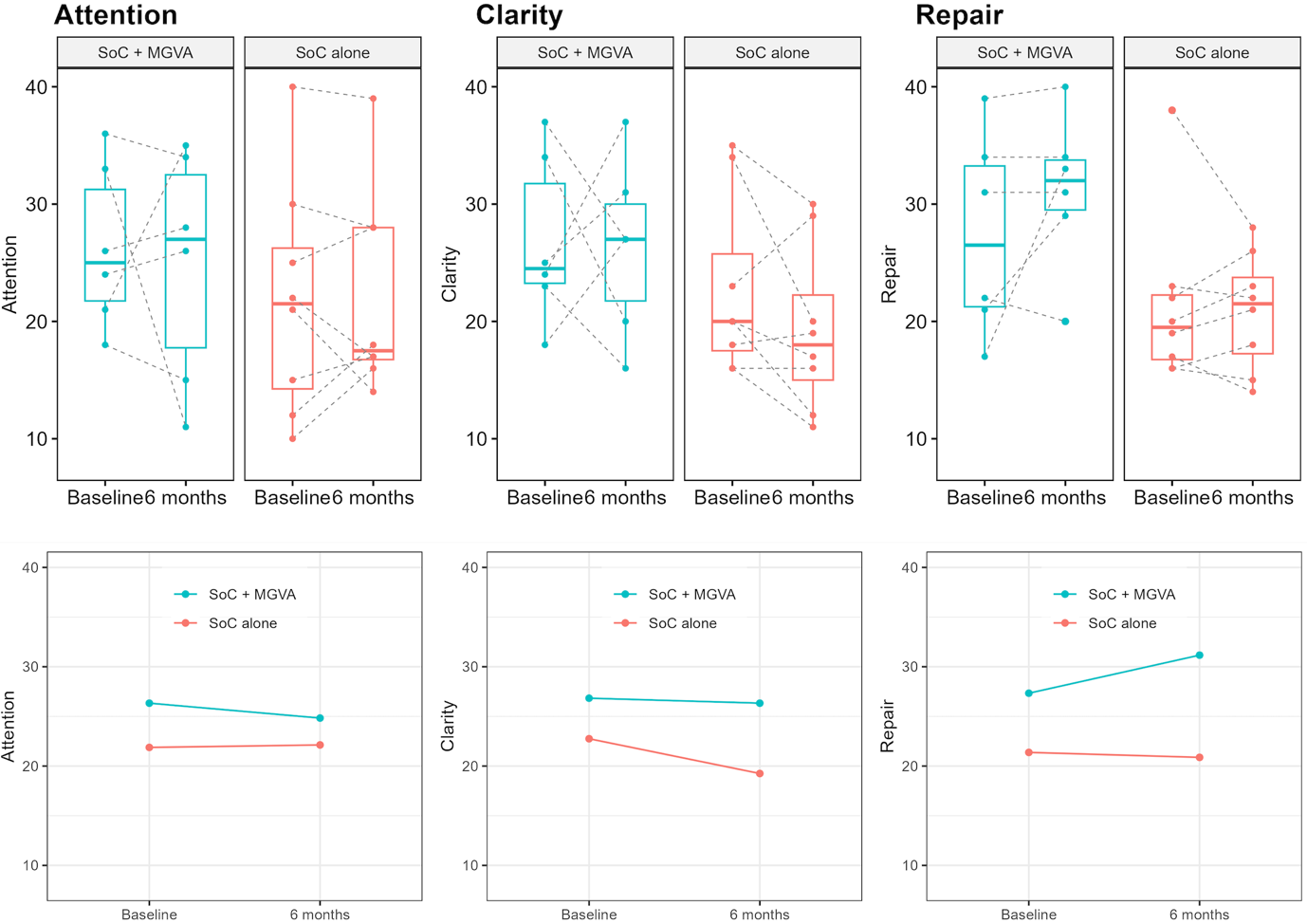
**
