# Supplementary material for: Feasibility of a multidisciplinary group videoconferencing approach for chronic low back pain: a randomized, open-label, controlled, pilot clinical trial (EN-FORMA)
Source: BMC Musculoskelet Disord. 2023 Aug 9;24:642. doi: 10.1186/s12891-023-06763-6 (PMC10410913; doi:10.1186/s12891-023-06763-6)
Supplement: Supplementary file 7 — Additional file 7: Supplementary Material 7. Mental wellbeing evaluated by the WEMWBS. [file 12891_2023_6763_MOESM7_ESM.docx]

**Supplementary Material 7:** Mental wellbeing evaluated by the WEMWBS

|  | **Baseline** | | **6 months** | |
| --- | --- | --- | --- | --- |
|  | **Experimental (SoC + MGVA)** | **Control (SoC alone)** | **Experimental (SoC + MGVA)** | **Control (SoC alone)** |
| Total Score, Mean (SD) | 43.3 (9.63) | 49.4 (10.3) | 45.8 (11.3) | 41.3 (9.43) |

**SoC:** Standard of Care; **SD**: Standard Deviation. Note: 1 missing value from the SoC group

**
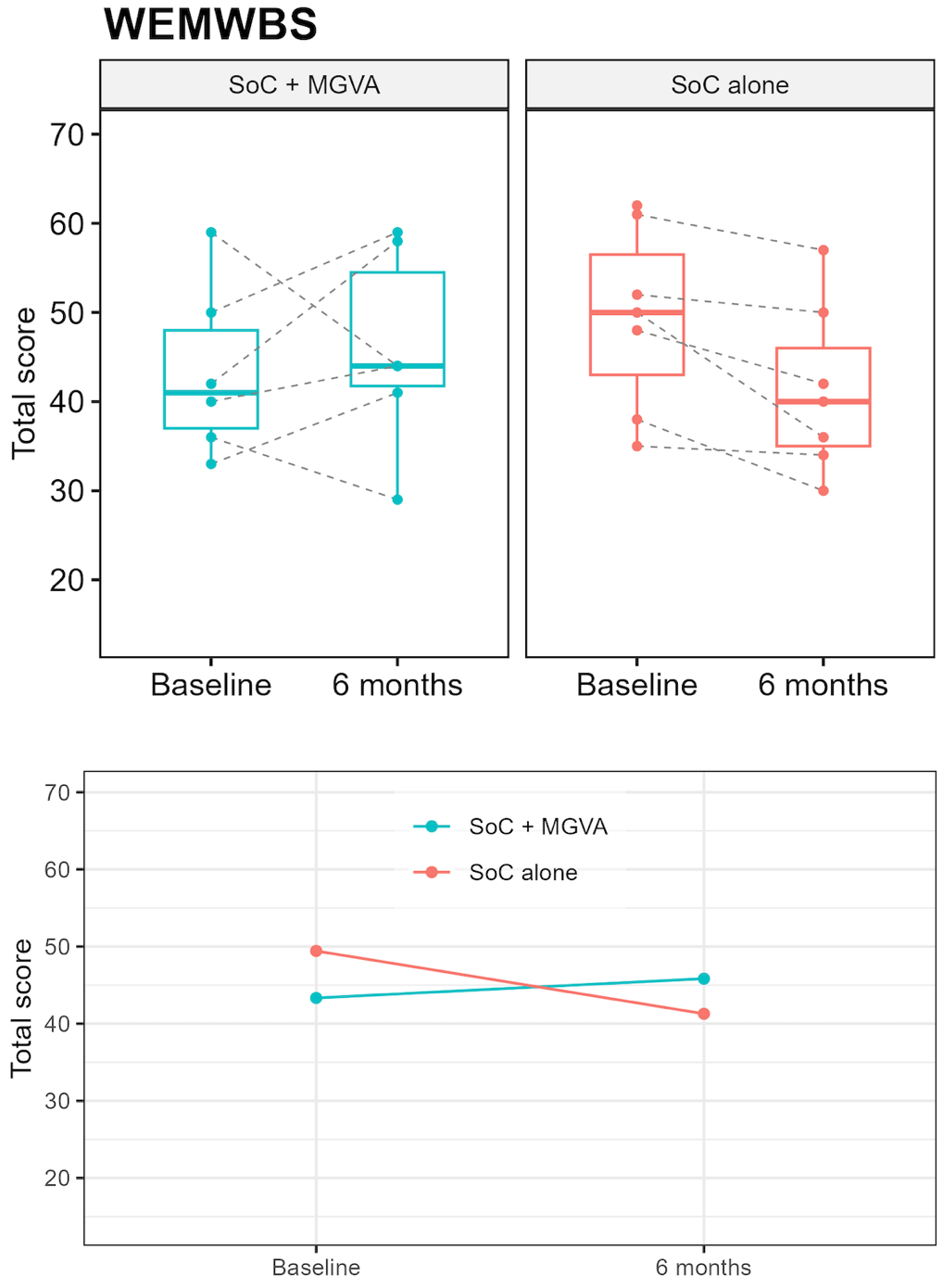
**
