# Supplementary material for: Feasibility of a multidisciplinary group videoconferencing approach for chronic low back pain: a randomized, open-label, controlled, pilot clinical trial (EN-FORMA)
Source: BMC Musculoskelet Disord. 2023 Aug 9;24:642. doi: 10.1186/s12891-023-06763-6 (PMC10410913; doi:10.1186/s12891-023-06763-6)
Supplement: Supplementary file 8 — Additional file 8: Supplementary Material 8. Level of estimated social support evaluated by the Oslo-3 scale. [file 12891_2023_6763_MOESM8_ESM.docx]

**Supplementary Material 8:** Level of estimated social support evaluated by the Oslo-3 scale.

|  | **Baseline** | | **6 months** | |
| --- | --- | --- | --- | --- |
|  | **Experimental (SoC + MGVA)** | **Control (SoC alone)** | **Experimental (SoC + MGVA)** | **Control (SoC alone)** |
| Total Score, Mean (SD) | 9.83 (1.60) | 8.88 (2.70) | 9.83 (1.94) | 8.75 (2.92) |

**SoC:** Standard of Care; **SD**: Standard Deviation.

**
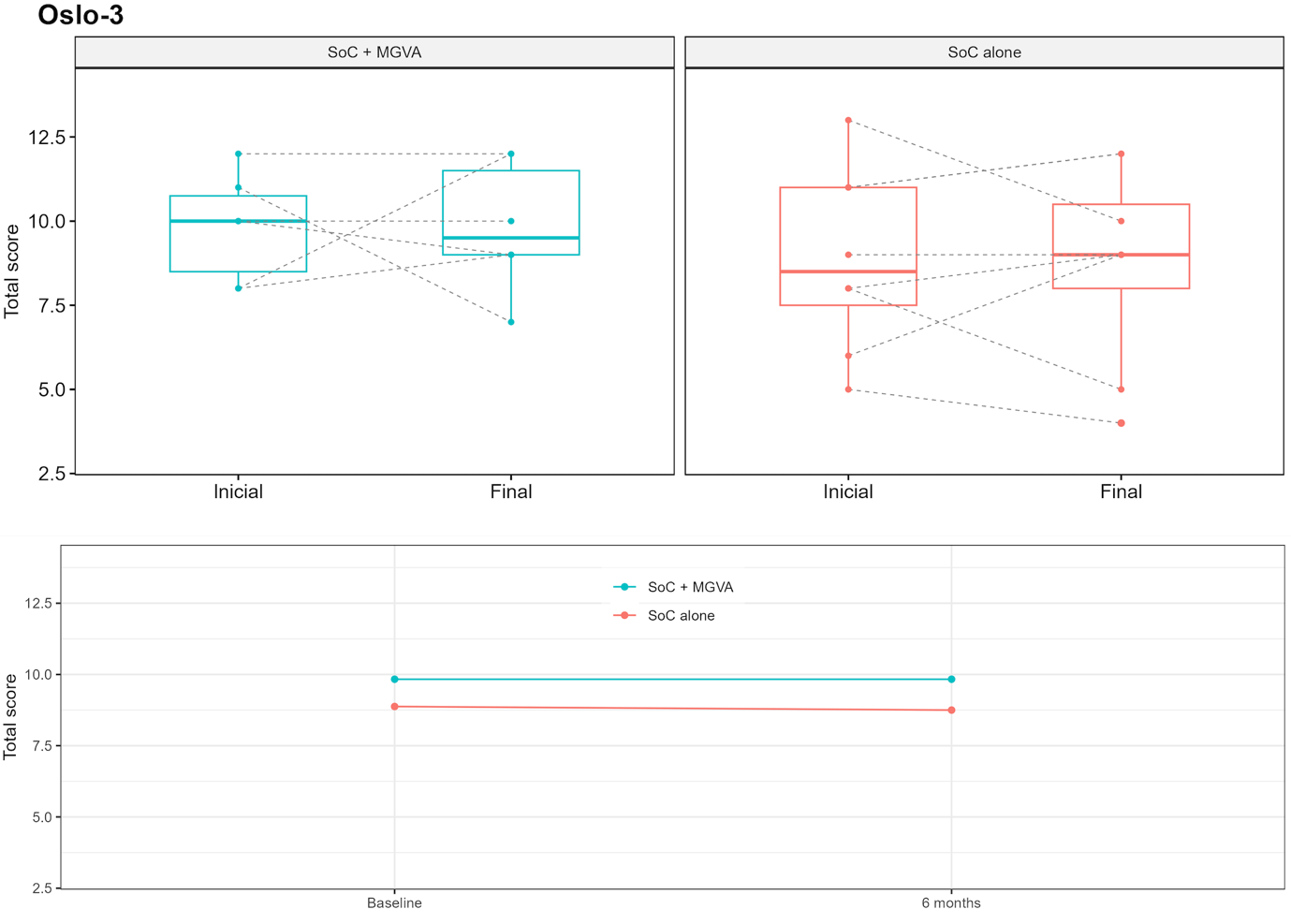
**
