# Supplementary material for: Feasibility of a multidisciplinary group videoconferencing approach for chronic low back pain: a randomized, open-label, controlled, pilot clinical trial (EN-FORMA)
Source: BMC Musculoskelet Disord. 2023 Aug 9;24:642. doi: 10.1186/s12891-023-06763-6 (PMC10410913; doi:10.1186/s12891-023-06763-6)
Supplement: Supplementary file 9 — Additional file 9: Supplementary Material 9. Model patient information sheet and other related documentation given to participants and authorized surrogates. [file 12891_2023_6763_MOESM9_ESM.docx]

**Supplementary Material 9.** Model patient information sheet and other related documentation given to participants and authorized surrogates

**PATIENT INFORMATION SHEET**

Study Title:

“**FEASIBILITY OF A MULTIDISCIPLINARY GROUP VIDEOCONFERENCING APPROACH FOR CHRONIC LOW BACK PAIN (EN-FORMA)”**

**Principal Investigator:** Dr Iago Garreta-Català

Orthopedic Surgery and Traumatology Department

We would like to inform you about a research study in which you are invited to participate. The study has been approved by a Clinical Research Ethics Committee in accordance with current legislation, Law 14/2007 on Biomedical Research.

We intend to provide you with the correct and sufficient information so that you can decide whether or not to participate in this study. Therefore, read this information sheet carefully, and feel free to ask us in case of any doubts. Additionally, you can consult with anyone you deem appropriate.

You should know that your PARTICIPATION in this study IS VOLUNTARY and that you may decide NOT to participate. If you choose to participate, you can change your decision and withdraw consent anytime, without having to give any explanations whatsoever and without this affecting the medical care you are entitled to.

**Introduction:** Chronic low back pain is a common problem in our society. Approximately 20% of the population suffers from it, and half have some degree of disability. People who suffer from it see their lives affected on many levels, and the problems that derive from the pain can end up contributing to its worsening.

Since the early 1980s, the characteristics of the behavior and social environment of patients with chronic low back pain have been studied. It has been shown that some reactions to pain, as well as certain interpersonal relationship aspects, can difficult the recovery project.

Many scientific articles have analyzed the efficacy of different treatment options for chronic low back pain. Some have shown that surgery may not improve the symptoms. On the other hand, group therapy with a combination of physiotherapy exercises and psychology and social work sessions (multidisciplinary approach) has shown promising results.

This study aims to assess the feasibility of a MULTIDISCIPLINARY GROUP VIDEOCONFERENCING APPROACH FOR CHRONIC LOW BACK PAIN. This approach will consist of 8 group videoconferencing sessions of physical rehabilitation/physiotherapy and psychosocial intervention offered as an integrated program for patients who have depleted non-surgical treatments. We believe that patients who participate in this program may improve their quality of life globally, as well as the degree of daily physical activity, disability due to low back pain, anxiety, and coping strategies for chronic pain.

**Benefits:** Your participation in this study could positively impact your daily life, improving your strategies to deal with chronic low back pain and increasing the available evidence on the efficacy of the multidisciplinary approach to treating low back pain. Your participation in the study will not change your current follow-up by other medical experts. In case is scheduled during the study, and you decide to go through with it, you will automatically be withdrawn from the study.

**Study Procedures**: A total of 20 participants are expected to be included in this study: 10 for the group to which the sessions will be offered, which will be called "multidisciplinary approach," and 10 for the control group. If you decide to participate, you will be interviewed by a physiatrist, a psychologist, and a social worker on separate days. At these visits, we will recheck that you meet the criteria to participate in the study, and demographic and disease data will be collected. After each visit, you will be sent an email with a link to fill in the questionnaires (8 in total).

If you are assigned to the control group, you will only have to fill in the questionnaires.

If you are assigned to the multidisciplinary focus group, the link to join the first group session will be emailed to you. Group sessions will take place on Tuesdays at 9 am and will take place using the free Google Meet® app that requires no download or installation. You will be required to create a Google account if you do not have one.

The duration of each session will be 2 hours, with the first part of 45 minutes of physiotherapy, a relaxation exercise of 15 minutes, and the second part of 1 hour of psychosocial intervention. You will need to wear comfortable clothes and have a yoga mat.

After finishing the last session, both the patients in the multidisciplinary approach group and those in the control group will be sent a new email with a link to fill out the most important questionnaire (called "quality of life"). Four months later, all participants will meet again with each therapist (individually) for reassessment. All will be sent a new link to fill out the eight questionnaires for the last time.

The **possible risks** are the same as those of physiotherapy sessions carried out in routine clinical practice to treat chronic low back pain and are minimal. If your low back pain worsens, we recommend you go to the emergency room for evaluation, just as if you had a worsening without participating in the study.

The main drawback you could have is not noticing a significant improvement in your symptoms or how to deal with them after finishing the sessions. The objective of the multidisciplinary approach is not to eliminate the pain but to improve the strategies that the patient has to deal with it and thus make it more tolerable and less disabling.

If you agree to participate, we also want to ask you for your commitment to the study and that you do not fail to come punctually to the visits or connect to the group sessions if it is not for a cause of force majeure. We also want to remind you that for the group sessions to be beneficial for everyone, it is essential that they take place in an atmosphere of respect and listening. If any patient has a disrespectful attitude that interferes with group therapy, they will be asked to leave the study and stop connecting to the sessions.

The promoter of the study is responsible for managing the financing of the study. You will not have to pay for study-specific tests. Your participation in the study will not entail any additional cost to your usual clinical practice.

**Personal data protection:** In compliance with Organic Law 3/2018 General on Data Protection and Guarantee of Digital Rights, the data collected for the study will be identified by a code so that it does not include information that can identify you, and only your study doctor/ collaborators will be able to relate said data with you and with your medical history. Therefore, your identity will not be revealed to anyone except in case of a medical emergency or legal requirement. The treatment, communication, and transfer of personal data of all participants will be in accordance with the provisions of this law.

Access to your personally identifiable information will be restricted to the study doctor and collaborators, health authorities, the Research Ethics Committee, and personnel authorized by the sponsor, when necessary to verify the study data and procedures, but always maintaining confidentiality of the same in accordance with current legislation. The data will be collected in a research file that is the responsibility of the institution and will be processed within the framework of their participation in this study.

The appropriate measures will be adopted to guarantee the protection of your privacy and will not allow your data to be crossed with other databases that could allow your identification.

In accordance with what is established by the data protection legislation, you can exercise the rights of access, rectification, opposition, and cancellation of data. Also, the rights to limit the processing of data that is incorrect, to request a copy, or to have it transferred to a third party (portability). You can exercise the aforementioned rights by contacting the main researcher or the DPD of the institution FUNDACIÓ TIC SALUT SOCIAL (dpd@ticsalutsocial.cat). In addition, you have the right to file a claim with the Catalan Authority for Data Protection if you consider that your data protection rights have been violated.

If you decide to withdraw consent to participate in this study, no new data will be added to the database, but data already collected will be used.

In accordance with current legislation, you have the right to be informed of the relevant data for your health that is obtained in the course of the study. This information will be communicated to you if you wish; In the event that you prefer not to be informed, your decision will be respected.

If you need more information about this study, you can contact the principal investigator, Dr Iago Garreta-Català.

Orthopedic Surgery and Traumatology Department of the Bellvitge University Hospital, on the 10th floor of the main building.

Telephone number: 93 260 75 72.
