# Supplementary material for: Feasibility of a multidisciplinary group videoconferencing approach for chronic low back pain: a randomized, open-label, controlled, pilot clinical trial (EN-FORMA)
Source: BMC Musculoskelet Disord. 2023 Aug 9;24:642. doi: 10.1186/s12891-023-06763-6 (PMC10410913; doi:10.1186/s12891-023-06763-6)
Supplement: Supplementary file 10 — Additional file 10: Supplementary Material 10. Model consent form and other related documentation given to participants and authorized surrogates. [file 12891_2023_6763_MOESM10_ESM.docx]

**Supplementary Material 10.** Model consent form and other related documentation given to participants and authorized surrogates

***INFORMED CONSENT FORM***

**Study Title:**

““**FEASIBILITY OF A MULTIDISCIPLINARY GROUP VIDEOCONFERENCING APPROACH FOR CHRONIC LOW BACK PAIN (EN-FORMA)”**

**Study Code:** HUB-COT-ENFORMA-2021

I, (given name and surname) __________________________________________________ have read the information sheet that has been given to me.

I have been able to ask questions and have received sufficient information about the study.

I have spoken with (investigator’s given name and surname) ____________________________________

I understand that my participation is voluntary and that I can withdraw from the study:

1. Anytime I want.
2. Without having to give any explanations whatsoever.
3. Without this affecting the medical care I am entitled to.

I will receive a signed and dated copy of this informed consent document.

I freely give my consent to participate in the study.

________________________ __________________________

Participant Signature Investigator Signature

Date: ____/____/____ Date: ____/____/____
